# Acid or base? How do plants regulate the ecology of their phylloplane?

**DOI:** 10.1093/aobpla/plab032

**Published:** 2021-06-10

**Authors:** Kadeem J Gilbert, Tanya Renner

**Affiliations:** Department of Entomology, The Pennsylvania State University, 501 Agricultural Sciences and Industries Building, University Park, PA 16802, USA

**Keywords:** Malvaceae, phylloplane pH, phylogenetic comparative methods, plant–insect interactions, plant–microbe interactions, plasma membrane H^+^-ATPases

## Abstract

Plants interface with and modify the external environment across their surfaces, and in so doing, can control or mitigate the impacts of abiotic stresses and also mediate their interactions with other organisms. Botanically, it is known that plant roots have a multi-faceted ability to modify rhizosphere conditions like pH, a factor with a large effect on a plant’s biotic interactions with microbes. But plants can also modify pH levels on the surfaces of their leaves. Plants can neutralize acid rain inputs in a period of hours, and either acidify or alkalinize the pH of neutral water droplets in minutes. The pH of the phylloplane—that is, the outermost surface of the leaf—varies across species, from incredibly acidic (carnivorous plants: as low as pH 1) to exceptionally alkaline (species in the plant family, Malvaceae, up to pH 11). However, most species mildly acidify droplets on the phylloplane by 1.5 orders of magnitude in pH. Just as rhizosphere pH helps shape the plant microbiome and is known to influence belowground interactions, so too can phylloplane pH influence aboveground interactions in plant canopies. In this review, we discuss phylloplane pH regulation from the physiological, molecular, evolutionary, and ecological perspectives and address knowledge gaps and identify future research directions.

## Teaser

Plants alter external environmental conditions in many ways. Well-known is the fact that roots alter the pH of the surrounding soil. However, less appreciated is the fact that plants also alter pH levels on their leaf surfaces. Our review explores this little-known topic; we discuss variation in leaf surface pH across a diversity of plants, the physiological ways by which plants can modify leaf surface pH and the importance of this trait to ecological interactions, including those with microbes and insects. This topic presents many unanswered questions awaiting future work, with relevance to agriculture as well as wild ecosystems.

## Introduction

In a vivid analogy, Vacher *et al*. (2016) deftly dispelled any perception of leaves as featureless two-dimensional planes with an enlightening shift in perspective: ‘Had bacteria eyes, their view of the leaf surface would not be smooth at all. It would look like a jungle in which epicuticular wax crystals form a rough terrain, veins are grooves, stomata and hydathodes are cracks and craters, and trichomes and fungal hyphae are trees and vines’. The aerial surfaces of plants, collectively called the phyllosphere, has been studied with increasing intensity since the 1950s, particularly in relation to its importance as a habitat for harmful and beneficial biota in an agricultural context (Dickinson 1976). To this aim, the topography of the peaks, valleys and craters of the phyllosphere have been mapped with tools such as SEM imaging. More and more, the creatures roaming (e.g. mites, protozoa, motile bacteria) and growing out of (e.g. fungal hyphae, colonies of algae or Actinobacteria) this landscape are being closely examined as well. Thus, it is perhaps surprising that the features of the aquatic component of the phyllosphere (or ‘phyllotelma’, as coined by Doan and Leveau (2015))—water droplets like lakes or seas, moisture spreading over the surface perhaps like rivers and waterfalls—has not received as much attention. In studies of leaf anatomy and physiology, there is often an implicit assumption that leaves are normally dry most of the time, but this may be untrue; wetness is a condition that may constitute a significant portion of the lifetime of a leaf (Dawson and Goldsmith 2018)—especially if ‘micro-wetness’ is considered as well (Burkhardt and Hunsche 2013). Consequently, the pH levels of moisture in the phyllosphere can be a trait of much importance to the microbiology of aerial plant surfaces as well as the physiology of the plant.

**Figure 1.**
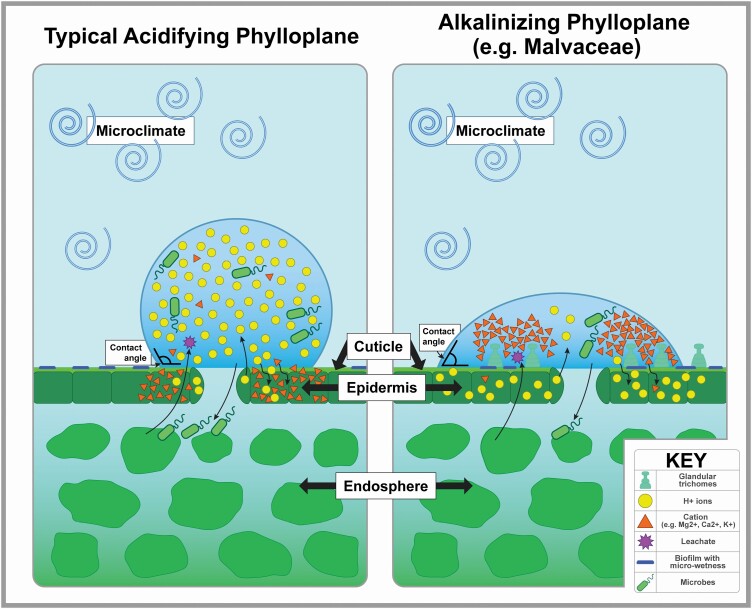
Water droplets interfacing with the leaf surface, displaying the morphological and physiological features relevant to probable mechanism(s) of phylloplane pH regulation. Representation of probable mechanism of phylloplane acidification, as in the mild acidification observed in most species (but also, the same physiological processes may be exaggerated to achieve hyper-acidification such as in carnivorous plants). In this case, the excretion of protons (H^+^) from guard cells and other epidermal cells outpaces H^+^ absorption through the cuticle. Epidermal cells may potentially absorb cations as well (as in *Sphagnum*). (Left) Representation of probable mechanism of phylloplane alkalinization, such as in Malvaceae. In this case, special glands excrete cations (e.g. Mg^2+^, Ca^2+^ and K^+^), and the absorption of H^+^ through the cuticle outpaces H^+^ excretion. (Right) In this figure, we also illustrate the difference between poorly wettable surfaces (high contact angles, left) and highly wettable surfaces (low contact angles, right). At a longer timescale, nutrients and metabolites may leach out of leaf tissues and affect the pH; however, this process is likely too slow to significantly influence the short-term alkalinization/acidification that is the focus of this review. We also allude to the likely influence of phylloplane pH regulation on the ability of exogenous microbes to survive and invade the leaf via stomata, and also its influence on the continued survival of already-established microbes on the phylloplane (such as biofilm-forming bacteria), which persist in droplets of micro-wetness in seemingly dry portions of the leaf surface. Illustration credit: Abraham Cone.

The impact of how plants may regulate the external pH is well-recognized for roots in the rhizosphere (Gerendás and Ratcliffe 2002; Hinsinger *et al*. 2003), but far less so for leaf surfaces, the phyllosphere. The phyllosphere comprises several layers: the inner portions of a leaf cross-section collectively known as the endosphere, as well as the outer portion, roughly from the epidermis and outwards (Fig. 1). This outermost layer is the phylloplane, which is the portion of the phyllosphere that most directly interfaces with the external environment, i.e. the living cells of the epidermis as well as the cuticle (Vacher *et al*. 2016). Even compared with internal leaf pH, phylloplane pH has been largely neglected. The relative paucity of phylloplane pH studies is readily apparent in the literature (Table 1). It is important to note that many of the studies that mention the word ‘pH’ may not include pH as a key topic (e.g. a study that isolates and cultures epiphytic bacteria may report the pH of the culture medium, yet not include data on the actual pH of the plant surface in question), so these numbers are likely over-inflated, further emphasizing the discrepancy between measurements of belowground and aboveground plant pH. Interestingly, there is also a stark paucity of ‘rhizoplane pH’ papers relative to ‘rhizosphere pH’ papers, just as ‘phylloplane pH’ is fewer in number than ‘phyllosphere pH’ (Table 1). Considering that the rhizosphere is defined as the layer of soil most under the influence of the root, extending away from the plant at some variable distance at the scale of millimetres (Hinsinger *et al*. 2003), while the rhizoplane is precisely the outermost layer of root touching that soil, this may point to an overall lack of attention to the most precise zone of plant–environment interface. However, the discrepancy in rhizosphere vs. rhizoplane search results may alternatively be due to authors opting to use only the broader term (‘-sphere’), rather than an actual lack of data on the precise zone of root-soil interface. In any case, regardless of the nature of the ‘-sphere’ vs. ‘-plane’ gaps, the ‘rhizo-’ vs. ‘phyllo-’ gap in pH studies remains large.

**Table 1.** Results of Web of Science search for Topic: ‘[Query]’ AND Topic: ‘pH’. Accessed March 2020.

Query	Rhizosphere	Rhizoplane	Phyllosphere	Phylloplane
No. of hits	3461	79	66	21

In this review, we will be focussing specifically on pH levels occurring on the phylloplane. We review the literature that has reported phylloplane pH levels and discuss what is known regarding the physiology of active phylloplane regulation and its consequences for plant ecology, especially noting numerous gaps in knowledge. While the topic of phylloplane pH has been the focus of very few studies, and has many unanswered questions (see Box 1), we provide information on the possible physiological/molecular underpinnings of variation in the trait, its taxonomic variation and possible evolutionary origins, and its ecological consequences to symbiotic organisms. We combine our review of the literature with original, phylogenetically informed analysis of the data to gain novel insights, and provide the botanical community with concrete hypotheses and future research directions for this topic. It is our intent to draw attention to this oft-overlooked plant trait, which has many implications for a plant’s ecological associations, including interactions with herbivores, pathogens and beneficial microbes. Much of the existing data on phylloplane pH comes from an agricultural context, so these implications extend to crop health and growth as well, including mitigating the damaging effects of acid rain on leaf tissues.

Box 1. **Some examples of the many unanswered questions that can be posed regarding the topic of phylloplane pH, including causes and consequences**

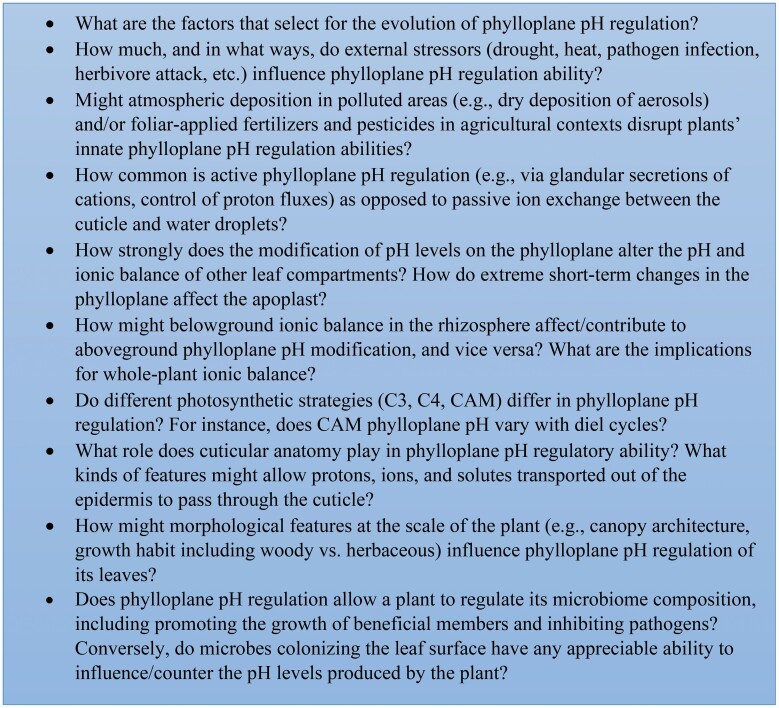

## Physiology and Possible Mechanisms of Short-term Phylloplane pH Modification

### Physical, chemical and anatomical features

The processes by which roots mediate changes in rhizosphere pH is well-studied. For instance, alkalinizing soil in response to excess anions or acidifying soil in response to excess cations (Hinsinger *et al*. 2003). The physiology of pH regulation is also well-studied in the context of internal pH, particularly intracellular pH, such as how cytoplasmic and vacuolar pH are maintained at ~7.5 and ~5.5, respectively (Smith and Raven 1979). Also, cell wall (apoplast) acidification has a known role in the growth of roots as well as aboveground parts, in connection with the ‘acid growth hypothesis’ (Van Volkenburgh and Davies 1983; Hejnowicz 1992; Yu *et al*. 2000; Visnovitz *et al*. 2012, 2013)—on the other hand, the leaf apoplast alkalinizes in response to biotic and abiotic stress (Geilfus 2017).

The earliest documented evidence that plants can differentially alter phylloplane pH comes from Oertli *et al*. (1977). The authors revealed the rapid changes (in a span of seconds to minutes) that occur in droplets introduced to a leaf surface, changes which occur in species-specific ways. In coffee (*Coffea arabica*) and common bean (*Phaseolus vulgaris*), the pH of a drop of deionized water (initial pH = 7.0) introduced to the leaf immediately drops to ~6. This short-term acidification matches the observation that the phylloplane is typically slightly acidic in most plants (Dickinson 1976; Oertli *et al*. 1977; Harr *et al*. 1984). In contrast to the immediate response of the other species in Oertli *et al*. (1977), rather than falling, the droplet’s pH on cotton (*Gossypium hirsutum*) immediately rose to 10.6. Many past studies investigating how leaves respond to external pH changes were done in the context of acid rain. In these simulated acid rain studies, crop species and deciduous trees were documented to raise the pH of acidic water droplets on their leaves over time (e.g. raising pH 3.6 droplets up to 5.8, or pH 4.6 droplets up to 6.9), also exhibiting species differences in this ability (Gaber and Hutchinson 1988a; Musselman 1988). In the ensuing paragraphs, we discuss several reasons for how and why such pH changes occur. Most phylloplane pH studies used excised leaf discs or epidermal peels (Klemm *et al*. 1987; Hauser *et al*. 1993; Smalley *et al*. 1993), but we will focus our discussion on studies that examine phylloplane pH regulation *in vivo* in a whole plant context (i.e. using flat-tipped pH probes to measure pH changes on living leaves) as we are particularly interested in the potential for active regulation in natural conditions.

At the scale of hours, interactions between water droplets and the physical environment can partly explain changes in pH, namely evaporation changes the concentration of solutes in the droplet which could change pH (Gaber and Hutchinson 1988a). Also, compounds that leach out of the leaf over time likely influences the resultant pH at this time scale (Adams and Hutchinson 1984; Tukey Jr 1970) as would compounds excreted out of the apoplast via guttation (Singh 2016). It is notable that whole-leaf pH (i.e. the pH of the homogenized phyllosphere, including mixed phylloplane and endosphere components) can vary independently of the soil pH environment in which the plant grows, thus leaf pH appears to be largely a plant-driven trait (Cornelissen *et al*. 2011). Importantly, though, Smith *et al*. (1996) showed that the pH of the phylloplane may differ from that of the entire homogenized leaf, such as the case of cotton, where the phylloplane is alkaline (>9.0) despite the homogenized leaf being slightly acidic (5.9–6.4). Thus, it is particularly remarkable to consider the rapid short-term changes in phylloplane pH documented by studies such as Oertli *et al*. (1977). Gaber and Hutchinson (1988a) suggest the involvement of an H^+^/cation exchange process between phylloplane and water droplets. In the case of cotton (*G. hirsutum*, the most well-studied species in terms of phylloplane pH), phylloplane pH increases can be linked to special ‘hydathode-like’ glandular trichomes (i.e. resembling the water-secreting pores involved in guttation, in that these glands are connected to the vascular system) that excrete cation microcrystals, mostly Mg^2+^, but also some concentration of K^+^ and Ca^2+^ (Elleman and Entwistle 1982). Interestingly, the glandular structures that Elleman and Entwistle (1982) described from *G. hirsutum* can also be seen in *G. barbadense*, *Abutilon theophrasti* and *Sida spinosa*; thus, these glands are possibly a common feature throughout Malvaceae (Harr *et al*. 1991; Harr and Guggenheim 1995).

The adaptive function(s) of the alkalinizing trichomes of Malvaceae remains unresolved (we explore potential evolutionary explanations in the section on Potential Evolutionary Context). Harr *et al*. (1984) raised potential physiological explanations including the maintenance of internal osmotic pressure (functioning like salt glands) and water uptake from the atmosphere (like the hypothesized function of the salty excretion of the desert shrub *Nolana mollis* [Mooney *et al*. 1980]). However, Harr *et al*. (1984) noted that phylloplane alkalinization is not limited to Malvaceae species from arid environments—rather, they found alkalinization in Malvaceae species from a variety of habitats.

### Molecular underpinnings

Regarding possible molecular mechanisms of controlling the flux of protons on the phylloplane to regulate pH, a promising candidate to investigate are the plasma membrane H^+^-ATPases. This is a gene family found in all plants, which functions in pumping protons (H^+^ ions) out of the cell membrane (Gaxiola *et al*. 2007) (EC 7.1.2.1). Studies that have examined differential gene expression in response to simulated acid rain point to a possible role of H^+^-ATPases in responding to external changes in pH (Kim *et al*. 2009; Liu *et al*. 2012; Satoh *et al*. 2014; Liang *et al*. 2015; Zheng *et al*. 2017; Ren *et al*. 2018). Liang *et al*. (2015) showed that moderately acidic treatments (pH 3.5) resulted in the upregulation of plasma membrane H^+^-ATPase gene expression, which helped stabilize intracellular H^+^ concentrations, whereas highly acidic treatments (pH 2.5) resulted in the downregulation of plasma membrane H^+^-ATPase expression in concordance with membrane damage and destabilized intracellular H^+^ concentration. This mechanism of transporting excess H^+^ ions out of the cells when exposed to moderately acidic pH in the rain may be widely involved in the mechanism by which different plant species modify the external pH of leaf surfaces. It is also worth noting that guard cells generally excrete protons during the process of stomatal opening (Edwards *et al.* 1988); perhaps plant species can differentially moderate this source of ionic flux.

Gene expression studies examining pH modification in other plant tissues also point to the importance of H^+^-ATPase gene evolution, namely in the cases of hyper-acidification in *Citrus* fruits (Strazzer *et al*. 2019) and *Petunia* petals (Faraco *et al*. 2014; Li *et al*. 2016). Strazzer *et al*. (2019) determined the molecular mechanism of how mutations in a regulatory gene leads to changed expression and the disruption of the typical citrus fruit hyper-acidification—typical hyper-acidification being characterized by juice vesicles with high H^+^ concentration and low H^+^ permeability (Müller *et al*. 1996). In *Petunia*, two H^+^-ATPase genes, *PH1* and *PH5*, regulate flower colour by hyper-acidifying petal vacuoles (i.e. low or high vacuolar pH making the anthocyanins appear red or blue, respectively; Faraco *et al*. 2014; Li *et al*. 2016). Interestingly, the genes that determine citrus fruit acidity are homologs of the pH regulatory genes that determine petunia flower colour, as confirmed by BLAST and phylogenetic analyses (Strazzer *et al*. 2019). The *PH5* genes in *Citrus* and *Petunia* are in turn homologs of the *Arabidopsis* H^+^-ATPase gene *AHA10*; and furthermore, homologs of both *PH1* and *PH5* are present throughout the angiosperm phylogeny, as well as in some gymnosperms, moss, and algae (Li *et al*. 2016). This may suggest potential ease for disparate plants to evolve similar acidification traits, via convergent evolution.

Although these genes are expressed in the vacuoles (Li *et al*. 2016; Strazzer *et al*. 2019), they belong to the plasma membrane H^+^-ATPase gene family rather than the separate V-type vacuolar ATPase gene family (Gaxiola *et al*. 2007), more specifically, they fall within Subfamily III (Li *et al*. 2016). Given that these genes can evolve novel expression in disparate tissues (Li *et al*. 2016; Strazzer *et al*. 2019), it is not unreasonable to expect they could evolve increased expression in the leaf epidermis too. In fact, evidence of a similar mechanism in the context of phylloplane hyper-acidification comes from carnivorous plants. Tropical pitcher plants (*Nepenthes*) have been demonstrated to regulate the pH levels of their pitcher fluid by controlling the flux of H^+^ ions into or out of the pitcher wall, which is the adaxial surface of the modified leaf (Moran *et al*. 2010), and some studies reveal relatively high levels of H^+^-ATPase gene expression in *Nepenthes* pitchers (An *et al*. 2001; Fukushima *et al*. 2017) and those of other carnivorous plants with acidic leaf surfaces (Fukushima *et al*. 2017). While the adaptive function of hyper-acidification in carnivorous plants might differ from that of most other plants (i.e. its role in prey digestion), common mechanisms appear to be at play. Indeed, carnivorous plants can be useful models for revealing insights about the upper extremes of phylloplane acidification ability.

As the plasma membrane H^+^-ATPase gene family has an ancient origin (Gaxiola *et al*. 2007), relevant insights on its role in hyper-acidification can be also found outside of vascular plants and even outside of land plants. Most algae (e.g. Chlorophyceae, Trebeouxiophyceae) and cyanobacteria are known to alkalinize their growth media; this is a consequence of photosynthesis and absorbing CO_2_ from their surroundings that would otherwise form carbonic acid (Shiraiwa *et al*. 1993). This method of alkalinization does not require ATPase activity. Contrary to all other known algae, members of the extremophilic family Cyanidiophyceae *acidify* their external environment. These algae live in acidic hot springs at temperatures of 38–56 °C and of pH 0.5–4.0—no other photosynthetic organisms withstand this combination of extremes (Lowell and Castenholz 2013). Cyanidiophyceae strains can acidify their growth medium down to a pH as low as ~2.5 (from an initial pH of ~5) as they grow; this is achieved by an ATP-dependent H^+^ efflux (Lowell and Castenholz 2013). This again shows the importance of plasma membrane H^+^-ATPases for evolving extreme acidification.

H^+^-ATPase genes may have some role in phylloplane pH regulation, whether hyper-acidification or hyper-alkalinization. On the alkaline extreme of the spectrum, while the molecular underpinnings of Malvaceae phylloplane alkalinization has yet to be explored with gene expression analyses, one study used genomic data from four *Gossypium* species to compare their P-type H^+^-ATPase genes in the context of cotton fibre colour (Chen *et al*. 2018). The tetraploid species *G. hirsutum* and *G. barbadense* each contain roughly twice as many H^+^-ATPase genes as the diploids *G. raimondii* and *G. arboreum*. Of note, the young leaves of *G. hirsutum* are extreme alkalinizers, whereas those of *G. arboreum* are mild acidifiers like most plants (Harr *et al*. (1984); also see Taxonomic Variation section). Perhaps H^+^-ATPase gene copy number influences alkalinization ability in *Gossypium*. Of course, much more sampling is needed across the genus to compare phylloplane pH regulation between the diploids and tetraploids.

Further research is needed to determine the molecular underpinnings of phylloplane pH regulation in plants, but it would be valuable to examine H^+^-ATPases in plants that differ in the phylloplane pH levels they achieve, given the ubiquity of this gene family and the number of cases in which it has been implicated in the evolution of hyper-acidification thus far. A logical next step would be to study differential gene expression in an experimental context for species with known differences in phylloplane pH regulation, comparing how gene expression changes in response to external pH changes (as in *in vivo* simulated acid rain experiments). There may also be several other pertinent genes involved, such as those that code for RALF (Rapid Alkalinization Factor) proteins (Felix *et al*. 1993; Sharma *et al*. 2016).

## Taxonomic Variation and Possible Evolutionary Context

### Algae

In investigating the early evolution of phylloplane pH regulation, it may be important to consider the aquatic algal ancestors of land plants. Whereas pH regulation by land plant leaves may be limited to a thin layer of moisture, algae living in an aquatic medium have a much more constant, larger external chemical environment to contend with—in this case, pH regulation may both be easier to achieve and possibly more consequential (effects on an aquatic environment that extends further beyond the leaf or cell). Indeed, all algae are capable of modifying the pH of their external environment, alkalinizing, or in rare cases, acidifying (see Physiology section) the water surrounding their cells/photosynthetic organs (Shiraiwa *et al*. 1993). Thus, the phylloplane pH regulation mechanism(s) may have ancient origins, with the necessary machinery being retained after the transition to land.

### Bryophytes

While bryophyte ‘leaf’ (thallus) surface pH has not been directly measured to our knowledge, mosses exhibit cation exchange ability. This has been especially well-documented for *Sphagnum* (Clymo 1963, 1964, 1984). Interestingly, while it was long believed that *Sphagnum*’s cation exchange ability was responsible for the significant acidification of bog water (e.g. *Sphagnum fusca* found to lower pH from 7.2 down to 5.9 within months) (Granath *et al*. 2010), one study finds that sphagnum cation exchange ability does not significantly differ from that of other mosses found from typically more alkaline fens, and thus found an alternate, physical explanation for bog acidification during fen-bog succession (Soudzilovskaia *et al*. 2010). Regardless of the extent to which moss cation exchange influences their larger scale external environment, the presence of this ability in bryophytes suggests that the mechanism(s) for leaf surface cation exchange may predate the origin of vascular plants. It would be valuable to directly measure phylloplane pH levels in bryophytes to better understand the small-scale changes that may occur as a result of the cation exchange process.

### Vascular plants

Overall, the phylogenetic coverage of phylloplane pH studies has been fairly limited. Phylloplane pH studies have largely focussed on agriculturally important plants, such as tomato (*Solanum lycopersicon*), beet (*Beta vulgaris* ssp. *vulgaris*), radish (*Raphanus sativus*), celery (*Apium graveolens*), spinach (*Spinacia oleracea*) and bean (*P. vulgaris*) (Adams and Hutchinson 1984; Musselman 1988). A handful of studies have examined woody trees (Gaber and Hutchinson 1988a, b), including *Cornus canadensis* and *Acer spicatum*. Thus, the data overwhelmingly comes from angiosperms. To our knowledge, no studies have directly measured pH levels on the leaf surfaces of gymnosperms; however, several studies indicate that acid rain causes increased foliar cation leaching in conifers (Huttunen *et al*. 1990; DeHayes *et al*. 1999). On the other hand, one throughfall study suggests that conifers lack the buffering capacity of broad-leaf trees: while throughfall pH was higher than precipitation pH for the deciduous forest site (during the growing season), suggesting some level of neutralization by the leaves, throughfall pH did not significantly differ from precipitation pH for the coniferous forest site over the three year study (Shibata and Sakuma 1996). To our knowledge, nothing has been reported on phylloplane pH regulation for lycophytes, ferns, or early-diverging angiosperms (*Amborella*-Nympheales-Austrobaileyales grade), meaning that how widespread the trait is across vascular plants is not currently known.

### Angiosperms

Much of the data on angiosperm phylloplane pH comes from an agricultural context. Multiple studies have shown that cotton, in contrast to most plants, is characterized by a highly alkaline phylloplane (Oertli *et al*. 1977; Young *et al*. 1977; Harr *et al*. 1980, 1984; Elleman and Entwistle 1982; Smith *et al*. 1996). Harr *et al*. (1984) further demonstrate that an alkaline pH appears to be characteristic of the mallow family Malvaceae as a whole, with multiple species across the family exhibiting the trait (note: the adaptive function of this trait is unknown, but the authors posited a role in pathogen defence as one possibility, see Ecological Relevance section). Malvaceae shows variation in phylloplane pH regulation, however. While nearly all the species examined strongly alkalinize the surfaces of their mature leaves, some species only mildly acidify their young leaves (roughly ranging from 6.5 down to 5.5, as is typical of most plants) before switching to alkalinizing the mature leaves (hereafter referred to as ‘age-dependent alkalinizers’). The ‘age-independent alkalinizers’, which alkalinize leaves of all stages, include *G. hirsutum*, *G. herbaceum* Kumpta, *Hibiscus trionum*, *Kitaibelia vitiifolia* and *Malva verticillata*, while the age-dependent alkalinizers include *G. arboretum* and *H. manihot*. All of the species listed here thus far exhibit concordant pH traits between adaxial and abaxial surfaces; however, the following species do not. *H. moscheutos* and *M. silvestris* have age-dependent alkalinization on their adaxial surfaces, but age-independent alkalinization on their abaxial surfaces. *Abelmoschus esculentus* also has age-independent alkalinization on its abaxial surface, but notably maintains a neutral pH (7.0, i.e. no change in pH relative to the distilled water used for measurements) on its adaxial surface independent of age. *Anoda cristata* again has age-independent alkalinization on the abaxial surface, but it is unique for exhibiting age-independent *acidification* on the adaxial surface (pH of 5.7, 6.3 and 6.8 for young, medium and old leaves, respectively). As was previously shown for *G. hirsutum* (Elleman and Entwistle 1982), all alkaline Malvaceae surfaces are associated with Mg^2+^, K^+^ and Ca^2+^ cation excretions (Harr *et al*. 1984). The reason(s) behind these interspecific and developmental differences in alkalinization remains unclear. Intraspecific differences between adaxial and abaxial surfaces are particularly puzzling. It would be interesting to examine the effect of leaf age on phylloplane pH in species outside of Malvaceae as well.

Outside Malvaceae, alkaline leaf conditions can be found in specialized halophytic plants, namely the genus *Tamarix* (Tamaricaceae, Caryophyllales). These evergreen shrubs and trees grow in highly saline soils and excrete excess minerals from the soil out of their salt glands, leading to the formation of an alkaline (mean pH ± SD = 8.5 ± 0.2) magnesium-rich dew covering their scale-like leaves at night when it is humid (Qvit-Raz *et al*. 2008). More specifically, *Tamarix aphylla* exhibits an alkaline phylloplane, whereas *T. nilotica* and *T. tetragena* do not, instead being neutral (Finkel *et al*. 2011). However, within *T. aphylla*, the phylloplane is only alkaline in certain locations and neutral in others, thus phylloplane alkalinity appears to be more heavily influenced by soil properties than as in the case of Malvaceae (Qvit-Raz *et al*. 2012).

The characteristically alkaline surfaces of Malvaceae, particularly *G. hirsutum*, represents one extreme of phylloplane pH regulation. The other extreme can be found in the highly acidic pitchers of tropical pitcher plants (*Nepenthes*), which are notable for being able to reach and maintain highly acidic pH in the fluid, in some species as low as pH 1 (Bittleston 2018; Saganová *et al*. 2018; Gilbert *et al*. 2020). In general, carnivorous plants must acidify their leaf surfaces to facilitate enzymatic activity of the digestive enzymes that are released in response to prey capture (Juniper *et al*. 1989; Ellison and Adamec 2018). For example, for the Venus flytrap (*Dionaea muscipula*), the initial pH of the excreted digestive fluid is ~4.3 and is then subsequently acidified to ~3.4 (Escalante-Pérez *et al*. 2011; Schulze *et al*. 2012). The pH inside the bladder traps of different *Utricularia* species range from 4.2 to 7.2, but is most typically around 5.1 (Sirová *et al*. 2003, 2009; Šimek *et al*. 2017). Like *Nepenthes* pitchers, *Utricularia* bladders host commensal/symbiotic organisms (Šimek *et al*. 2017) that must be adapted to the fluid pH levels set by the plant. Notably, carnivorous plants occur in 5 disparate angiosperm orders, thus the evolution of acidic trap leaf surfaces is a case of convergent evolution.

### Potential evolutionary context

**Figure 2.**
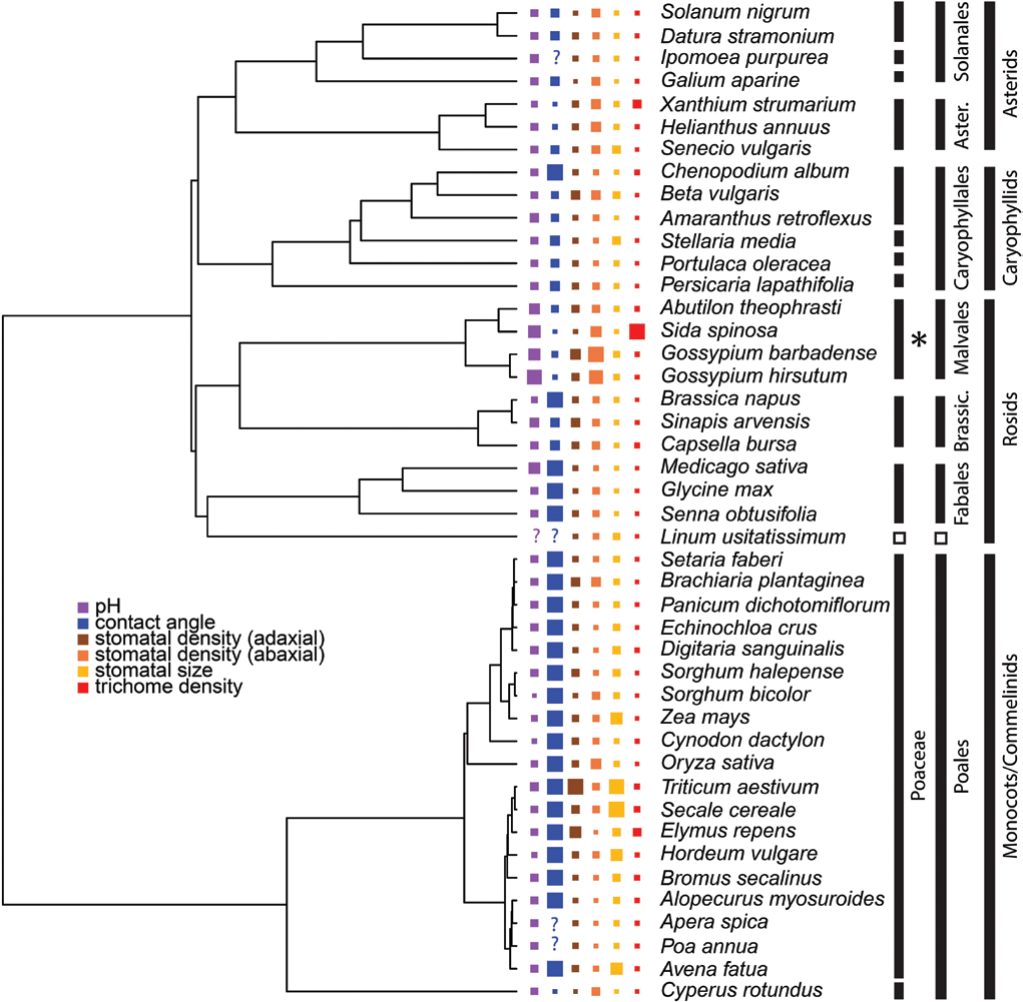
‘Leaf Surfaces’ series data (‘Leaf Surfaces of Major Crops’ (Harr and Guggenheim 1995) and ‘Leaf Surfaces of Major Weeds’ (Harr *et al*. 1991)) in a phylogenetic context. The figure displays the values for pH, contact angle (without surfactant), adaxial and abaxial stomatal density, (adaxial) stomatal size and trichome density with the values scaled to their relative magnitude (bigger squares equals larger values). ‘?’ indicates missing data in the original source. Bars to the right of the tree indicate taxonomic information (at level of family, order, and above). The asterisk indicates the placement of the characteristically alkaline family, Malvaceae. The open boxes in the Rosids section indicate the sole species in Linaceae/Malpighiales, which lacks pH data.

While it is difficult to conclude much about the evolution of pH regulation across plants due to limited sampling, it is still possible to conduct preliminary analyses and find possible trends within angiosperms using available data. The most comprehensive source of phylloplane pH data comes from a pair of agricultural publications ‘The Leaf Surface of Major Crops’ (Harr and Guggenheim 1995) and ‘The Leaf Surface of Major Weeds’ (Harr *et al*. 1991). These two publications represent a set conducted by the same research group using directly comparable methods on a variety of plants: a total of 45 species in 15 families, covering 9 angiosperm orders including both eudicots and monocots. Although phylloplane pH regulation was not analysed in a phylogenetic context within these works, their phylogenetic breadth allows for a preliminary look into trait evolution (Fig. 2, our methods are described in the ensuing paragraphs).

To obtain our phylogeny, we used the Zanne *et al*. (2014) supertree, for which almost all species in the dataset were included. We used the ‘phytools’ package (Revell 2012) to trim the supertree; this and all analyses were done using R v. 3.5.0 (R Core Team 2013). Species names here reflect the Zanne *et al*. (2014) taxonomy; some different synonymous species names were used in the original ‘Leaf Surfaces’ entries (*Agropyron repens = Elymus repens*, *Cassia obtusifolia = Senna obtusifolia*, *Polygonum lapathifolium = Persicaria lapathifolia*). The supertree lacked the hybrid species *Triticale* (*Triticum* × *Secale*), so for the purpose of representing it in this analysis, we attributed the ‘Leaf Surfaces’ data from *Triticale* to its non-represented parent species *Secale cereale*. *Xanthium orientale* was also absent from the supertree, so we applied the data on *X. orientale* to the closely related *X. strumarium*. However, we note that excluding the *Triticale* and *X. orientale* data does not significantly affect results of our analyses.

The ‘Leaf Surfaces’ series included phylloplane pH data as a graph of pH level recorded over a 20-min time period. To contextualize the analysis in terms of the short-term phylloplane pH regulation that is the focus of this paper, we used the pH level of the leaf surface at minute 2 of their measurement as our pH trait. Note that the authors only recorded pH levels from the adaxial surface, thus our discussion on correlates of pH level focuses on adaxial traits.

In addition to phylloplane pH, the authors of the ‘Leaf Surface’ series recorded contact angle (measuring the point of contact between leaf surface and water droplet: lower angles indicate greater wettability, see Fig. 1), percent polar/non-polar compounds in the cuticle (for ‘Weeds’ book only), and included scanning electron microscope (SEM) images of the adaxial and abaxial leaf surfaces for each species at 70×, 350× and 3500× magnification displayed as 70 × 70 mm square frames. From this SEM data, we calculated estimates of stomatal size, stomatal density and trichome density. For stomatal size, we used the 350× image and measured the length and width (from the outer edge of one guard cell to the next) of each stomatal aperture in the frame with a standard metric ruler, recorded in millimetres. We took the average length and width for all stomata in each frame, converted those two stomatal dimensions to their corresponding actual dimensions in microns based on the magnification information provided, and then calculated our final estimate of stomatal size using the formula for the area of an ellipse—this was done separately for the abaxial and adaxial photographs for each species.

To estimate the abaxial and adaxial stomatal densities for each species, we multiplied the stomatal size value by the number of stomata in each 350× frame to get the total area of the SEM image that is covered by stomata, and then divided this number by the total area of the square frame (converted to the actual area in microns: 14 000 μm) to get the final stomatal density value for the abaxial and adaxial surfaces for each species. For trichome density, we simply counted the total number of (non-glandular) trichomes in each 70× frame. We found no difference to our analyses whether we used adaxial or abaxial trichome counts or the sum of the two. Here we used the summed trichome counts.

We tested for phylogenetic signal in traits with Pagel’s lambda (Pagel 1999) using the ‘phylosig’ function in the ‘phytools’ package. We conducted phylogenetic generalized least squares tests using the ‘pgls’ function in the ‘caper’ package (Orme 2013).

We found that most of the traits we scored show significant phylogenetic signal, including pH (Pagel’s lambda = 0.72, *P* < 0.0001), contact angle (Pagel’s lambda = 0.94, *P* < 0.0001), percent polar/non-polar cuticular wax composition (Pagel’s lambda = 0.88, *P* < 0.0001, for either), adaxial stomatal size (Pagel’s lambda = 0.19, *P* = 0.02) and abaxial stomatal density (Pagel’s lambda = 0.65, *P* < 0.0001).

**Figure 3.**
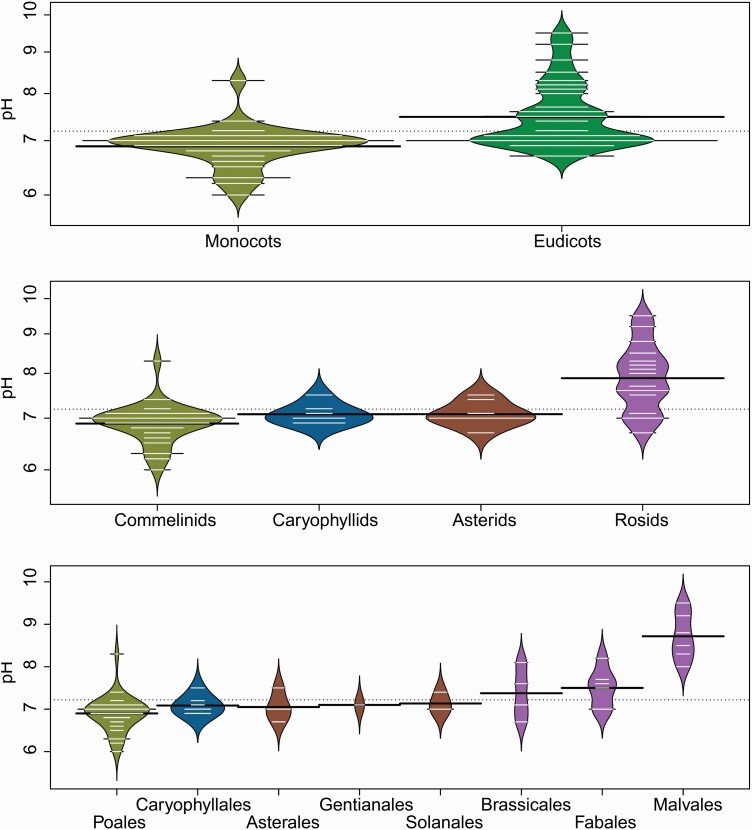
Beanplots displaying range of pH values from the ‘Leaf Surfaces’ dataset (Harr *et al*. 1991; Harr and Guggenheim 1995), at three taxonomic scales. The width of short white lines represents the number of species at each value; long black lines represent means for each taxon. Beanplots are colour-coded taxonomically: yellow = Monocots, green = Eudicots, blue = Caryophyllids, red = Asterids, purple = Rosids.

This dataset corroborates previous studies which show that Malvaceae exhibit unusually high phylloplane pH in relation to all other plants. Not only this, but also note that Rosids in general have a higher mean pH than other angiosperms here, as Fabales and Brassicales also have some slightly higher values compared with the Asterid, Caryophyllid and Commelinid clades (Fig. 3). Contact angle shows even clearer phylogenetic signal as almost all of the monocots included in the dataset have large contact angles (i.e. low wettability), in fact they are generally non-wettable with a contact angle of 180°. However, it should be noted that all of these species are grasses in the family Poaceae, and the one monocot with a lower contact angle is the only non-Poaceae monocot represented (the reed *Cyperus rotundus*, family Cyperaceae), thus while it may be possible to infer that Poaceae in general may have high contact angles, it is not possible to generalize this conclusion to monocots as a whole. Interestingly, this dataset also suggests that monocots have a lower mean pH than eudicots (Kruskal–Wallis χ ^2^ = 13.81, *P* < 0.001), not only driven by uniquely high pH values from within Malvales, but also by uniquely low values within Poales (Fig. 3), though note that this dataset does not include species with extremely low pH levels such as carnivorous plants.

We found that an increase in phylloplane pH level is strongly correlated with decreasing contact angle (pgls coefficient = −0.009, *P* = 0.0183) and increasing adaxial stomatal density (pgls coefficient = 2.49, *P* < 0.001). In other words, the species with the highest phylloplane pH are the most wettable and have the highest stomatal densities—in this case, this holds for all representatives of Malvaceae in the dataset. While glandular trichomes are linked to Malvaceae alkalinization (Elleman and Entwistle 1982) and one Malvaceae species (*S. spinosa*) had an exceptionally high trichome density, trichome density did not exhibit sufficient variance in this dataset to infer any general patterns. It is worth noting that the structure of the cuticle is another conceivable factor that can vary across species; however, very little data is available on the cuticular morphology of the species represented here (Riederer and Muller 2008). On the other hand, cuticular chemistry is represented here as percent polar or non-polar wax composition, although this was only available for ‘Leaf Surfaces of Major Weeds’. Using this subset, phylloplane pH has a positive correlation with percent non-polar (pgls coefficient = 0.005, *P* = 0.0125) or a corresponding negative correlation with percent polar (pgls coefficient = −0.005, *P* = 0.0125).

**Figure 4.**
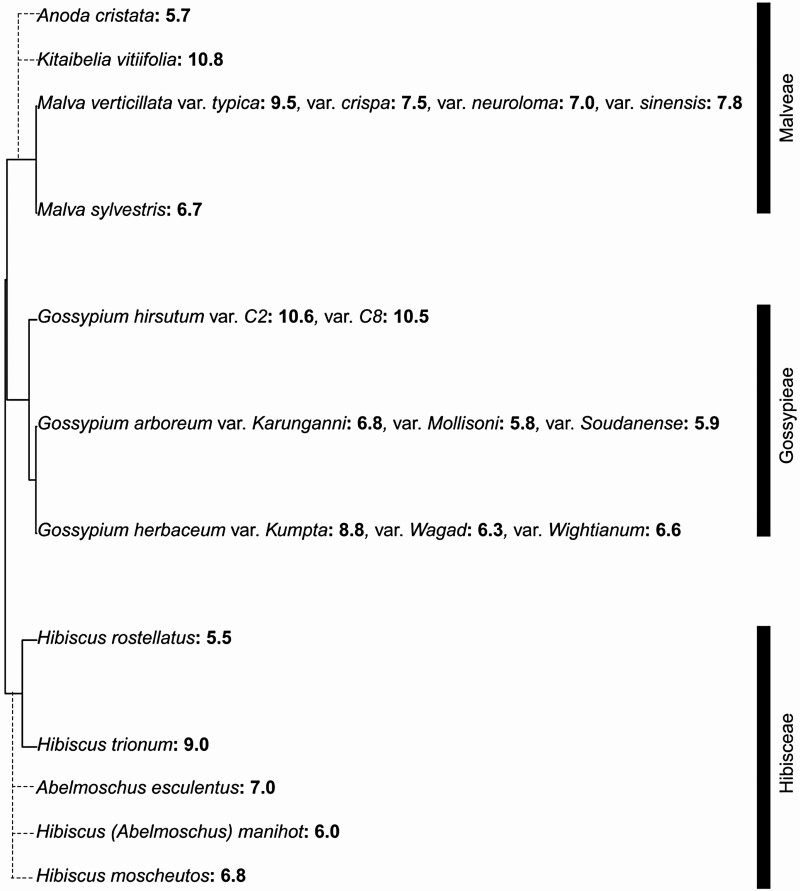
Data on Malvaceae phylloplane pH regulation from Harr *et al*. (1984) in a phylogenetic context. To obtain our phylogeny, we used the ‘phytools’ package (Revell 2012) in R v. 3.5.0 to trim the Zanne *et al*. (2014) supertree. Species that were not included in the Zanne *et al*. (2014) supertree (*Anoda cristata*, *Kitaibelia vitifolia*, *Abelmoscus esculentus*, *Hibiscus manihot* and *Hibiscus moscheutos*) are represented here using dashed line branches. These placements are based on taxonomic designations (i.e. classification of genera into tribes Malveae and Hibisceae, USDA 2020), and is meant only to display inclusion into one of three clades in the tree—topology and branch lengths for these additional species are arbitrary. Numbers in bold display the adaxial pH levels of young leaves from each species/variety. For species with multiple varieties, each separate variety is designated by ‘var.’

While we do see phylogenetic signal in trait variation here, with the highest phylloplane pH values restricted to the Malvaceae, we find some evidence of lability as well. For ‘Leaf Surfaces of Major Crops’ (Harr and Guggenheim 1995), the authors included data on pH for multiple varieties of certain species. The analyses we have thus far discussed only included a single variety per species, as other trait data (contact angle and SEM image data used for estimating stomatal and trichome traits) were only provided for the primary variety for each species. Most species have consistent pH data across varieties; however, a couple exceptions can be seen: whereas the primary representative(s) of their respective species exhibit the typical mild acidification in the 2-min period, the variety *Brassica napus* ‘Bienvenu’ (Brassicaceae) and *Hordeum vulgare* ‘Triton’ show alkalinization more akin to Malvaceae (pH at 2 min of 8.1 and 8.3, respectively). These may be outliers, or evidence that phylloplane pH regulation can rapidly evolve. Further evidence of the latter possibility can be found in (Harr *et al*. 1984), which we also place into a phylogenetic context here (Fig. 4). The phylogeny suggests that each of the three represented lineages has lost or gained (age-independent) alkalinization at least once independently. Additionally, some species with data for multiple varieties reveals potential intraspecific lability; for instance, different *Gossypium herbaceum* varieties either display age-independent (‘Kumpta’) or age-dependent (‘Wagad’ and ‘Wightianum’) alkalinization.

## Ecological Relevance

As noted previously, much of the data on phylloplane pH regulation comes from simulated acid rain studies, accordingly it is known that one major ecological function of phylloplane pH relates to buffering against the external environment. Species that are better able to buffer and raise the pH of acidic droplets were also found to show less tissue damage in response to those droplets (Adams and Hutchinson 1984; Musselman 1988). While phylloplane pH regulation is clearly involved with protection from abiotic stressors, the trait may play a role in a number of biotic interactions as well.

An anti-herbivore function for alkaline phylloplane pH was demonstrated in cotton, where varieties with higher pH were more distasteful to caterpillars (Navon *et al*. 1988). Not only this, but tobacco whitefly has been found to be highly discriminating in the pH of its potential food sources, with a strong preference for leaves or artificial diet of pH 6.0–7.25, and they have the remarkable ability to discriminate pH level to a precision of 0.25 units (albeit this refers to internal leaf pH in this case) (Berlinger *et al*. 1983). Given variation in plant phylloplane pH and the ability of at least some insects to discriminate leaf pH levels, it seems likely that phylloplane (and phyllosphere more generally) pH may have a wider influence on plant–herbivore interactions. It is, however, unclear whether phylloplane pH has any impact on arthropods outside of a trophic context. While this is unknown for the phylloplane, work examining a different segment of the phyllosphere may help: some studies have examined the pH of bark (Everhart *et al*. 2008; Kӧhler *et al*. 2015; Zuo *et al*. 2016). Bark pH was shown to influence the species composition of slime mold (Myxomycetes) communities living on that substrate (Everhart *et al*. 2008); on the other hand, bark pH does not significantly influence arthropod community composition (Zuo *et al*. 2016).

In many other environmental contexts (e.g. soil, lakes, animal guts), pH is known to be a highly important factor for microbes, including within the rhizosphere where the pH is largely controlled by the plant (Gerendás and Ratcliffe 2002; Hinsinger *et al*. 2003). It is also worth noting that whole-leaf pH is now known to be highly important to litter decomposition belowground (Tao *et al*. 2019). Thus, given the highly specific pH requirements of bacteria and fungi, it is likely that a plant’s ability to regulate phylloplane pH can also help select for a particular microbial community composition, just as it is in the rhizosphere (Schoninger *et al*. 2012; Mendes *et al*. 2013). However, despite some mention of this hypothesis in the phylloplane pH literature (Oertli *et al*. 1977; Elleman and Entwistle 1982; Harr *et al*. 1984), this has not been empirically examined for the most part. However, one reason cotton phylloplane pH has received attention is that *Heliothis* NPV, a virus-derived foliar insecticide, is inactivated at high pH levels, thus reducing its effectiveness on cotton (Young *et al*. 1977).

Based on our phylogenetic analysis, we conjecture that high leaf wettability and stomatal density may have predisposed Malvaceae for phylloplane alkalinization. Highly wettable leaves means that water would be in contact with more of the leaf surface, and potentially have a longer residence time on the leaf after wetting events. It makes sense that leaves that repel water (and thus do not strongly interface with them) would be less able to manipulate the pH of that water. On that same token, it makes sense that leaves that interface more readily with water may face more selective pressure to manipulate the properties of that moisture contacting the leaf. We suspect that the plants’ interactions with microbes may be a major factor behind these results. Wet leaves increase the risk of infection by microbial pathogens (Kim *et al*. 2002; Kumar *et al*. 2004; Dawson and Goldsmith 2018). Furthermore, stomatal properties have been shown to influence how well pathogenic microbes enter the leaf—higher stomatal density, increased stomatal size, and higher stomatal conductance all predictably lead to increased infection risk (Ramos and Volin 1987; Mathur *et al*. 2013; Murray *et al*. 2016; Dutton *et al*. 2019). Furthermore, plants can adaptively respond to infection by reducing their stomatal density; this response is known for bacterial and fungal pathogens (Dutton *et al*. 2019), and more recently discovered to be a response to viruses as well (Murray *et al*. 2016). So, the combination of high wettability and high stomatal density makes sense as the favoured conditions for promoting the evolution of greater magnitude phylloplane pH modification in Malvaceae.

There is largely a lack of published studies specifically examining the effect of interspecific (or intraspecific) variation in phylloplane pH regulation on the microbiome. Some studies have noted the direct effects of acid rain on pathogens or mutualistic endophytes in leaves (Cheplick 1993; von Sury and Flückiger 1993). Studies of tropical pitcher plants (*Nepenthes*) have revealed a notable degree of interspecific variation in pH regulation within the genus, which can lead to differences in microbial community composition (Kanokratana *et al*. 2016; Bittleston *et al*. 2018; Gilbert *et al*. 2020). Like many leaf-associated communities, Proteobacteria dominate *Nepenthes* pitcher fluid bacterial communities. Decreasing fluid pH leads corresponds to increasing relative abundance of certain acidophilic taxa such as Acetobacteraceae, whilst most other taxa decrease in relative abundance. As a result, less acidic pitchers have higher alpha diversity at the community level (Gilbert *et al*. 2020)

The microbiome of the highly alkaline surfaces of *Tamarix* has also been examined, revealing communities comprised salt- and desiccation-tolerant extremophiles (e.g. *Halomonas*, *Marinococcus*, *Deinococcus*) similar to those found in soda lakes (Qvit-Raz *et al*. 2008, 2012; Finkel *et al*. 2011). One study found geography to be more important to *Tamarix* phylloplane community structuring than pH (or salinity); however, pH levels also differed between sites (Qvit-Raz *et al*. 2012)—this study did not examine multiple host species. Finkel *et al*. (2011) found species differences in *Tamarix* phylloplane pH; however, in this case the pronounced species differences in pH did not influence overall microbial community composition as much as geography. Even still, the influence of pH can be seen here at a smaller scale, as the alkaline *T. aphylla* lacked certain Proteobacteria found on the other two examined host species in the Mediterranean (Finkel *et al*. 2011).

## Conclusions and Future Directions

Despite the many gaps in knowledge, this review demonstrates that there are many reasons to pay more attention to phylloplane pH regulation. For instance, there are many far-reaching applications for agriculture. An increased understanding of the physiology of phylloplane pH regulation can lead to better understanding and combating the susceptibility of crops to acid rain. It is already known in the case of cotton that phylloplane pH levels can affect foliar-applied pesticides, so variation in phylloplane pH traits may have implications for foliar-applied sprays more widely, be they pesticides or fertilizers (Fernández and Brown 2013). Further, phylloplane pH may directly affect herbivorous insects as well as the community of microbes living on the leaf, which means there is potential for plants to regulate interactions with both pathogens and mutualists (such as plant growth-promoting methanogens or entomopathogenic fungi: Morris 2001; Thapa and Prasanna 2018). The potential impact of modifying leaf surface pH is not limited to rainy days and humid nights either, as the existence of micro-wetness means there is always moisture on the leaf to manipulate (Burkhardt and Hunsche 2013). Grinberg *et al*. (2019) recently discovered how microscopic leaf wetness is important for allowing bacterial survival on seemingly dry leaf surfaces.

We note that the vast majority of studies that have directly examined phylloplane pH thus far have focussed on crops or agriculturally relevant plants. However, the abovementioned ecological implications of the trait are likely equally important in natural systems, thus examining phylloplane pH regulation may yield insight into plant physiology, evolution and ecology at a much more fundamental level; so, it will be valuable to examine this trait in a wide variety of different environmental contexts, including wild plants. We recommend focussing on taxonomic breadth and conducting *in vivo* phylloplane pH measurements for a wide assortment of species. Collecting phylloplane pH data for gymnosperms, ferns and bryophytes would be especially useful for investigating the evolutionary history of the phylloplane pH regulation trait. While angiosperms are currently the source of all direct data on phylloplane pH, more studies are needed within angiosperms as well. We need a better understanding of baseline phylloplane pH levels from species that vary widely in phylogeny and ecology: for instance, sampling more woody plants, non-graminaceous monocots and non-agricultural forbs. Many questions remain regarding the full range of phylloplane pH variation, and the phylogenetic/physiological constraints to reaching the extremes. Is hyper-alkalinization largely limited to the family Malvaceae? Might alkaline surfaces be an important feature of salt-excreting halophytes other than *Tamarix*? Do any non-carnivorous plant phylloplanes approach the hyper-acidity of carnivorous plants? These questions all require broader sampling to answer.

Moreover, studies of phylloplane pH should measure other leaf surface features, including wettability and stomatal density. We predict that all species with extreme alkalinity or acidity should have high wettability, as well as exhibit higher than average stomatal densities. Relatedly, the glandular trichomes of Malvaceae require more attention. Can differences in alkalinization traits be linked to morphological/physiological differences in these glands? Future work should follow up on Harr *et al*. (1984), to examine what determines the difference between age-independent and age-dependent alkalinizers. Comparing gland densities between species/varieties with differing pH levels is one place to start. Experimental gene expression analyses may also be useful for this point. Peng *et al*. (2016) found that varieties of *G. hirsutum* that differ in salt-tolerance differ in how much salt can be excreted out of their glandular trichomes, which is in part regulated by H^+^-ATPase activity—this may be relevant for understanding the differences in alkalinization traits. The H^+^-ATPase gene family is of interest for examining the molecular basis of phylloplane pH regulation, perhaps both for alkalinization as well as acidification.

 Finally, in addition to future work investigating the evolution and physiology of phylloplane pH regulation, we see an opportunity for more ecological work, including further examining the role of phylloplane pH in interactions with herbivores and microbes. The microbial component is of special interest for future work. The ‘Leaf Surfaces’ series (Harr *et al*. 1991; Harr and Guggenheim 1995), the most comprehensive study focussed on phylloplane pH to date, was published in the early 1990s. Since then, there have been considerable advancements in sequencing technology and molecular techniques that now allow more detailed microbial investigations than before. Culture-independent sequencing techniques like metabarcoding (Baker *et al*. 2016) enable the simultaneous examination of the entire community of bacteria, fungi, protists, microscopic animals, archaea and viruses living on the phylloplane. Furthermore, metagenomics, metatranscriptomics and metabolomics approaches can complement community composition data with data on community function (Aguiar-Pulido *et al*. 2016). We recommend comparing the microbiomes of leaf surfaces from species with and without extreme phylloplane pH modification to jump-start knowledge of the importance of pH to aboveground microbes. To return to the perspective shift with which we began, the ‘phyllotelma’ is a waterscape much like a lake teeming with fish (microbes): just imagine how limited knowledge of limnology would be were data on such essential water properties like pH left unexamined.

## Data Availability

R code and data files associated with the phylogenetic comparative analysis will be made available on Penn State’s ScholarSphere (https://scholarsphere.psu.edu/) doi:10.26207/ycxk-8207.

## Notes

**Form & Function Chief Editor:** Kate McCulloh
